# The paradoxical rarity of a fruit fly fungus attacking a broad range of hosts

**DOI:** 10.1002/ece3.6585

**Published:** 2020-07-20

**Authors:** Camiel Doorenweerd, Sebastian Sievert, Walter Rossi, Daniel Rubinoff

**Affiliations:** ^1^ Department of Plant and Environmental Protection Sciences Entomology Section College of Tropical Agriculture and Human Resources University of Hawaii Honolulu HI USA; ^2^ Department of MeSVA Environmental Sciences Section University of L'Aquila Coppito Italy

**Keywords:** Ascomycota, *Bactrocera dorsalis*, biocontrol, Dacini, Laboulbeniales, Oriental fruit fly, Tephritidae

## Abstract

Understanding the factors that determine the realized and potential distribution of a species requires knowledge of abiotic, physiological, limitations as well as ecological interactions. Fungi of the order Laboulbeniales specialize on arthropods and are typically thought to be highly specialized on a single species or closely related group of species. Because infections are almost exclusively transmitted through direct contact between the hosts, the host ecology, to a large extent, determines the distribution and occurrence of the fungus. We examined ~20,000 fruit flies (Diptera: Dacinae) collected in Malaysia, Sulawesi, Australia, and the Solomon Islands between 2017 and 2019 for fungal infections and found 197 infected flies across eight different *Bactrocera* species. Morphology and 1,363 bps of small subunit (18S) DNA sequences both support that the infections are from a single polyphagous fungal species *Stigmatomyces dacinus*—a known ectoparasite of these fruit flies. This leads to the question: why is *S. dacinus* rare, when its hosts are widespread and abundant? In addition, the hosts are all *Bactrocera,* a genus with ~480 species, but 37 *Bactrocera* species found sympatric with the hosts were never infected. Host‐selection does not appear to be phylogenetically correlated. These results suggest a hidden complexity in how different, but closely related, host species vary in their susceptibility, which somehow limits the abundance and dispersal capability of the fungus.

## Graphical abstract

Understanding the mechanisms that determine infection rates will involve both study of host resistance as well as host ecology, as the fungal parasites depend on physical contact of the hosts to be transmitted. Ultimately, this can inform on a broader scale on how host–parasite density systems function in practice and can potentially be applied for pest control.

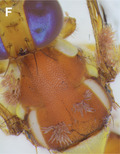

## 1 INTRODUCTION

Species distributions are often governed by abiotic, physiological, limitations (e.g., climate), as well as ecological interactions (Booth, 2017). The latter, in particular, can be highly dynamic and, in the special case of host‐parasite interactions, often part of an “evolutionary arms race” that can escalate into specialized interactions and ultimately phylogenetic co‐evolution (Joop & Vilcinskas, 2016; Winkler & Mitter, 2008). Alternatively, some parasites instead evolve to be generalists, which potentially opens up a larger niche and a corresponding distribution exceeding that of a single host species, but often with apparent trade‐offs resulting in reduced infection rates. The distributional limitations of parasites can be especially challenging to understand when hosts are plentiful but infection rates remain low. For fungi, a plausible limitation on their distribution and abundance is that their hosts have evolved a high level of resistance. Yet, entomopathogenic fungi are ubiquitous and some are known as ecologically important regulators of insect population densities (Pedrini et al., 2015), and an abundant host should be vulnerable to devastating waves of infections (Cory & Myers, 2009). Theoretic and practical frameworks at present have few answers in cases where there is a ubiquitous and abundant host, yet fungal infection rates remain low. Understanding how potentially devastating pathogens with broad access to an abundant host can remain rare is not only relevant for understanding broader host‐parasite interactions and their implications for the health of vulnerable populations, but also for controlling major agricultural pests which appear exempt from the reductive impacts of their parasite guilds.

Laboulbeniales (Ascomycota) includes around 2,100 described species of ectoparasitic fungi with a global distribution (Madelin, 1966; Rossi & Kirk‐Spriggs, 2011). They have evolved to specialize on arthropods, and typically infect only the cuticle, although some have more or less developed rhizoids that enter the host beyond the cuticle (Rossi & Feijen, 2018; Rossi & Kirk‐Spriggs, 2011; Rossi, Vávra, & Barták, 2019). The arthropod cuticle is the first line of defense against fungal infections and is strongly selected for this function (De Kesel, 1996; Hajek & St Leger, 1994; Pedrini et al., 2015), suggesting that a host‐pathogen arms race is likely. The entire life cycle of Laboulbeniales is completed on living arthropods, and dispersal occurs only through physical contact between viable hosts, often during mating (Haelewaters, Page, & Pfister, 2018; Pfliegler, Báthori, Wang, Tartally, & Haelewaters, 2018; Rossi & Kirk‐Spriggs, 2011) or in some situations through intermediate substrates, for example, in an ant nest (De Kesel, 1996). Laboulbeniales are typically thought to be highly specialized (De Kesel, 1996; De Kesel & Haelewaters, 2014), but some seem polyphagous (Rossi, Santamaría, & Andrade, 2013). Because dispersal depends on physical contact, and conspecific hosts physically interact at least during mating, it seems likely that there is an evolutionary advantage to specialization on a single host species. Many reports of polyphagy (i.e., more than a single host species) have not been substantiated using molecular methods, and it is possible that they form a complex of sibling fungal species without distinguishing morphological characters (Haelewaters, De Kesel, & Pfister, 2018). Some recent studies using DNA are revealing polyphagy, (Haelewaters et al., 2019; Haelewaters, De Kesel, et al., 2018), yet others reveal cryptic speciation on sympatric hosts (Haelewaters, De Kesel, et al., 2018). Knowledge of the distribution and host use of Laboulbeniales in general is fragmented (Rossi et al., 2013), but some groups have been studied in more detail and reveal a complex mix of co‐evolutionary patterns at different trophic levels. For example, hyperparasitic Laboulbeniales that infect flightless bat flies (Nycteribiidae and Streblidae) revealed co‐evolutionary phylogenetic associations between old world bat flies and their fungi but the phylogenetic relationships in neotropical fungal genera are more congruent with the roosting ecology of the bats, which determines where the infected bat flies come into contact with each other (Haelewaters, Page, et al., 2018). These results indicate that the dispersal of the fungi is completely passive and determined by the ecology of the hosts or superhosts. Thus, the distribution and host range of parasitic fungi is seemingly dictated by host ecology and distribution.

The fruit fly genus *Bactrocera* (Tephritidae: Dacinae) includes about 460 species, with the vast majority native to the (sub)tropical Oriental, Australian and Oceanian regions, and less than ten representatives in Africa (Doorenweerd, Leblanc, Norrbom, San Jose, & Rubinoff, 2018). The larvae of all species feed on fruit or flowers and at least 70 species are agricultural pests, invading large parts of the world (De Meyer et al., 2010; Vargas, Pinero, & Leblanc, 2015). The best‐known pest is the Oriental fruit fly *Bactrocera dorsalis* (Hendel), which feeds on over 300 fruits and vegetables (Allwood et al., 1999), ranges across Asia, has invaded all of Africa, much of the Pacific, and, more recently, entered southern Europe (Nugnes, Russo, Viggiani, & Bernardo, 2018; Stephens, Kriticos, & Leriche, 2007; Vargas et al., 2015). Although some of the pest *Bactrocera*, like the Oriental fruit fly, are highly polyphagous, the majority of species are more specialized and feed on one or a few closely related hosts. Adult *Bactrocera* are known to interact during courtship, mating and at suitable oviposition sites, but several species have also been documented pollinating flowers, for example *Bulbophyllum* orchids of section *Cheiri* (Tan, Nishida, & Toong, 2002). The orchids, in return, provide the flies with synomone chemicals which they incorporate in their mating pheromones, resulting in increased fecundity (Keng‐Hong & Nishida, 2005). *Bactrocera* flies are known to congregate at such orchid flowers, providing additional opportunity for physical contact and intra‐ and interspecific fungal transmission.

A species of Laboulbeniales that infects *Bactrocera* fruit flies, *Stigmatomyces dacinus* Thaxt., was described from Sarawak, Borneo (Thaxter, 1918). Since then, it has been reported in only one study, infecting an unidentified tephritid fruit fly from Sierra Leone, and on *Ceratitis cosyra* (Walker, 1849) and *Coelotrypes* cf. *vittatus* Bezzi 1923 from Kenya (Rossi & Leonardi, 2018). As part of ongoing research on the systematics of *Bactrocera* and related genera, we assessed the frequency of *Stigmatomyces* infections on multiple species in the group to address a series of questions relating to the occurrence and dispersal of ectoparasitic *Stigmatomyces* fungi on *Bactrocera* fruit flies. Laboratory studies on Laboulbeniales infection of the lesser housefly *Fannia canicularis* (Linnaeus) showed that infections are nonfatal (Whisler, 1968), but there is a general lack of field data on infection rates and modes of transmission. If the infection is of moderate cost to the host, it should be expected to increase in abundance as host populations are not severely impacted, especially if the host is abundant. To understand how the fungus interacts with *Bactrocera* and disperses, we surveyed for fungal infections among ~25,000 fruit flies in our collections and compared the fungal DNA of infected flies to (a) investigate whether different fly species in the same genus are infected by different fungal species, (b) estimate the biogeographic distribution of *S. dacinus*, (c) estimate the potential distribution of the fungus based on its realized niche, and (d) assess the impact the fungus might have on populations of the various species of *Bactrocera*.

## 2 MATERIAL AND METHODS

### 2.1 Sampling

*Bactrocera* flies were collected in the field using 120 ml pee‐cup bucket traps baited with male lures methyl eugenol, cuelure, and zingerone, which are specific to *Bactrocera,* and the related genera *Zeugodacus* and *Dacus,* joined together in the tribe Dacini. For further details on the trap design and implementation see Leblanc, Tora Vueti, and Allwood (2013). The lures attract different species; therefore, three traps were put out at each location with approximately two meters between them and trap captures were kept separate per lure. Sets of traps were typically placed every 150–500 m along a transect or, where possible, a loop trail. Traps were emptied after 2–7 days, and flies were placed directly into 95% ethanol. All samples were stored at −20°C until further study. Photographs of flies with fungal infections were taken using a Nikon D3100 camera mounted to an Olympus SZ10 stereo microscope, using the focus‐merge option in Affinity Photo 1.7.3 software to generate a larger focal plane.

Roughly, 20,000 fly specimens that we collected across Asia (Sabah, Malaysia; Sulawesi, Indonesia), Australia (Northern Territory and Queensland), and Oceania (Solomon Islands) during Dacini fruit fly surveys in 2017–2019 were systematically checked for the presence of ectoparasitic fungi. We found eight infected species in total. Five species had more than ten infected specimens, for which we went back to our collections prior to 2017 and examined all bulk samples present in the University of Hawaii Insect Museum (UHIM) collections for the presence of ectoparasitic fungi. A bulk sample consists of one or multiple specimens of a single species from a single lure trap, at a single locality. We studied 14 bulk trap samples for *Bactrocera aquilonis*, 25 for *Bactrocera decumana*, 82 for *Bactrocera kraussi*, 56 for *Bactrocera neohumeralis*, and 71 for *Bactrocera tryoni*, in total 248 traps and 2,442 flies (Tables S1, S2). The number of flies per trap ranged from one to 156, with an average of 9.8. We plotted distribution maps and box plots of the infection rates with Python 3.7 using various libraries, including Pandas, Numpy, Seaborn, and Basemap.

### 2.2 DNA extraction, PCR and sequencing

To extract the DNA from the fungus and avoid host DNA, we removed a bush of thalli using forceps, typically taking 20–100 thalli. In a few cases, less than 20 thalli were present and all were sampled. DNA was then extracted using a Qiagen DNeasy blood and tissue kit (Qiagen LLC), following the manufacturer's protocol. PCR reactions consisted of 13.3 μl of RedExtract Taq polymerase (Sigma‐Aldrich), 2.5 μl of each 10 μM primer, 5.7 μl of H2O, and 1.0 μl of template DNA. We amplified a section of the small subunit (SSU) 18S with primers NSL1 (5′‐GTA GTG TCC TCr CAT GCT TTT GAC‐3′) and NSL1 R (5′‐TGA TCC TTC TGC AGG TTC ACC TAC G‐3′) from Haelewaters, Page, et al. (2018). We were unable to amplify segments of 5′ large subunit (LSU) or internal transcribed spacer (ITS) with the primers published in Haelewaters, Page, et al. (2018) and Schoch et al. (2012). All amplifications were performed in a BioRad C1000 Thermal Cycler with initial denaturation at 94°C for 3:00 min; followed by 35 cycles of denaturation at 94°C for 1:00 min, annealing at 50°C for 0:45 min and extension at 72°C for 1:30 min; and final extension at 72°C for 10:00 min. Bidirectional Sanger sequencing was outsourced to Eurofins. We combined resulting chromatograms into consensus sequences using Geneious R10 (https://www.geneious.com) and deposited them in Genbank (accessions MN935796–MN935814; Table S3).

### 2.3 Phylogenetic analysis

We successfully obtained 19 *S. dacinus* SSU sequences from 22 fly specimens, representing all eight infected *Bactrocera* species. We aligned these with the 12 published *Stigmatomyces* SSU sequences from Haelewaters, Page, et al. (2018) and the five *Stigmatomyces* SSU sequences published in Goldmann and Weir (2018), resulting in an alignment with 36 sequences and 1,743 base‐pairs. We used IQ‐Tree 1.6.10 (Nguyen, Schmidt, von Haeseler, & Minh, 2015) to infer a maximum likelihood tree with 10,000 ultrafast bootstrap replicates and 10,000 Shimodaira–Hasegawa‐like approximate likelihood ratio test (SH‐like aLRT) replicates to estimate branch support. The IQ‐Tree integrated ModelFinder Bayesian information criterion (BIC) selected model of evolution was HKY+F+I. The resulting tree was visualized using FigTree 1.4.3 (https://github.com/rambaut/figtree/) and optimized for publication using Affinity Designer 1.7.3 (https://affinity.serif.com).

## 3 RESULTS

**FIGURE 1:**
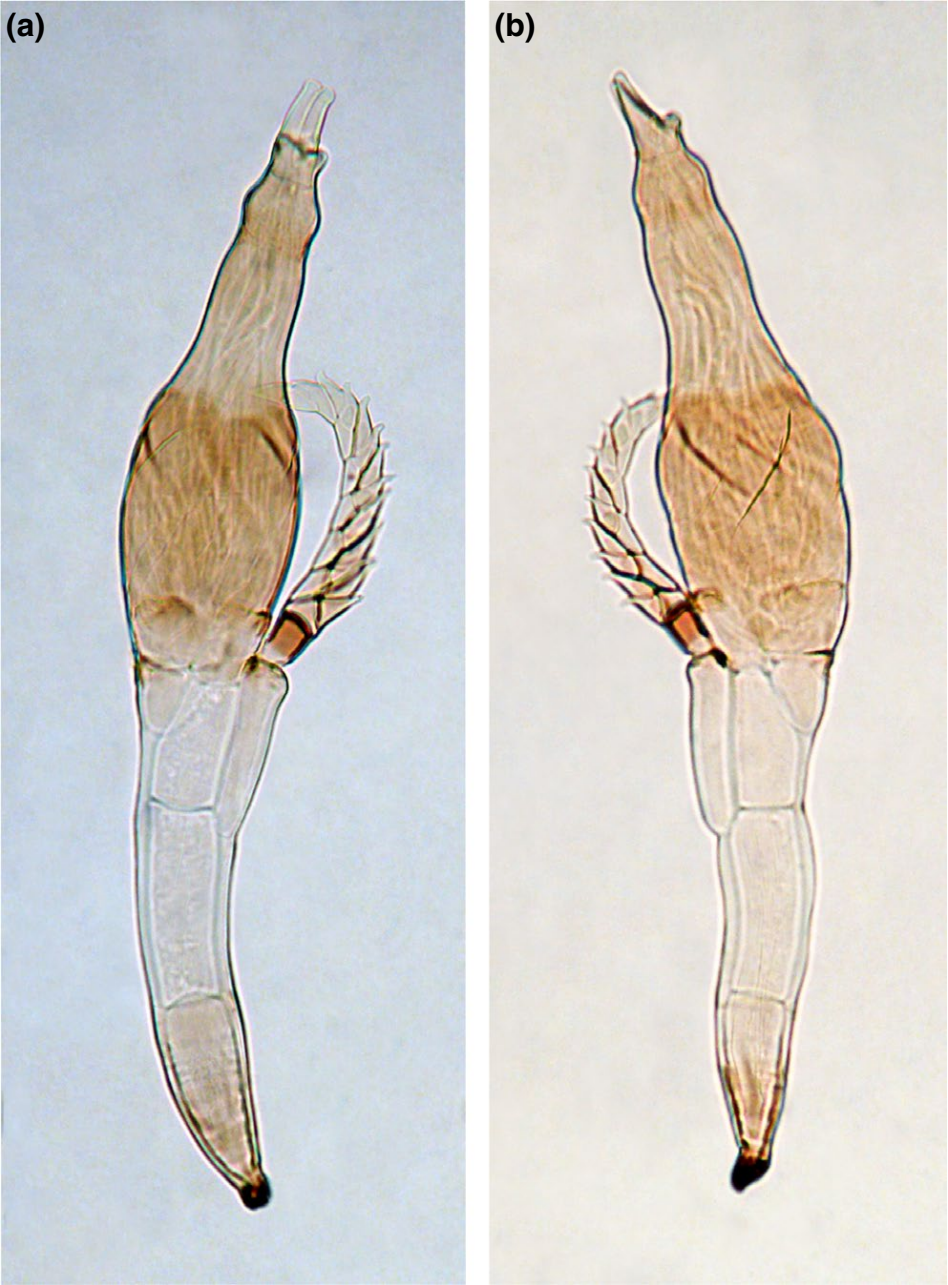
Two *Stigmatomyces dacinus* Thaxt. thalli, both ex *Bactrocera aquilonis* (Australia, Palmerston, 26–27.v.2017). (a) thallus with tip and apex in front view, length 0.305 mm, (b) thallus with tip and apex in lateral view, length 0.315 mm

**FIGURE 2:**
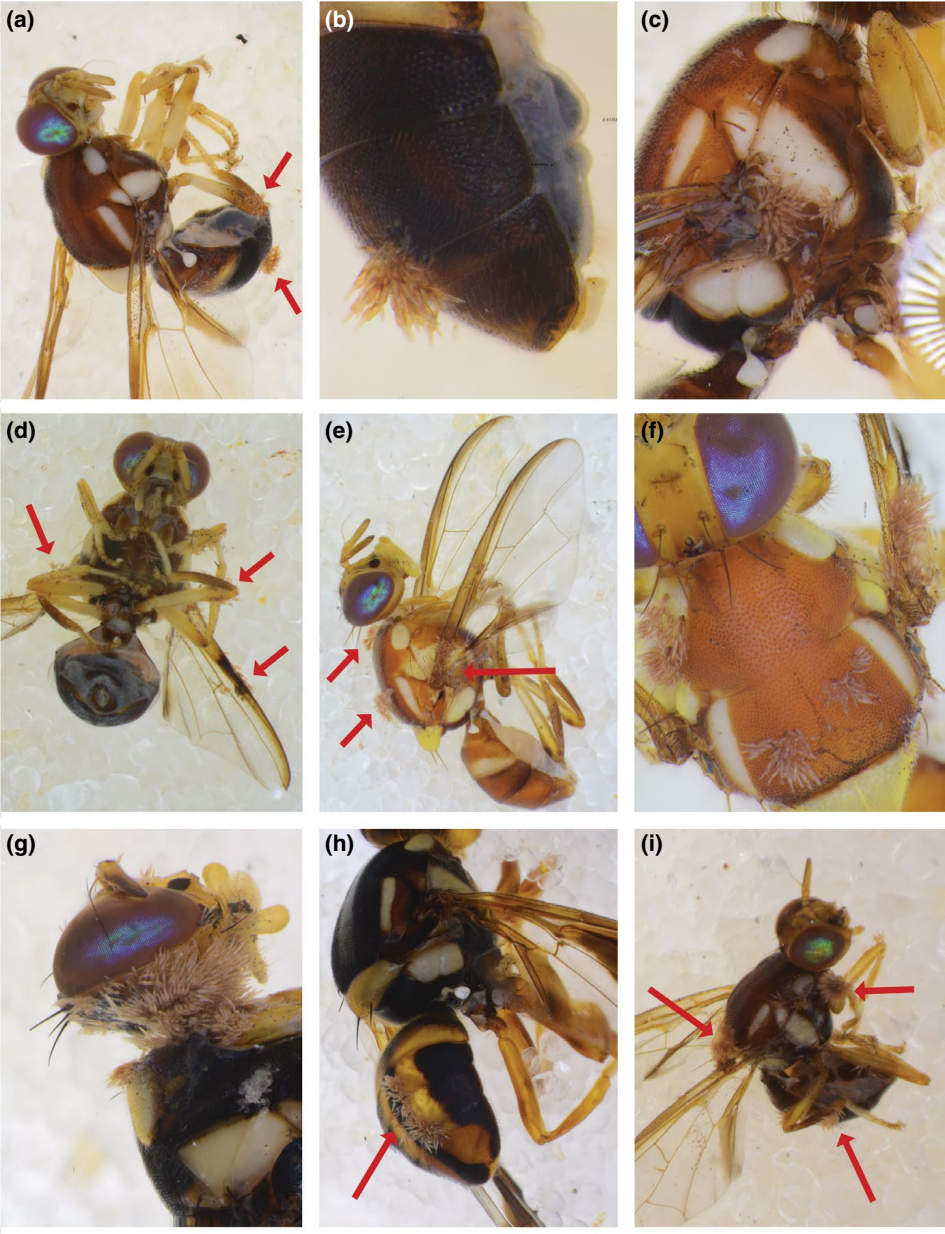
Overview of some of the different species and body parts that we found infected by *Stigmatomyces dacinus*. (a) Cluster of *S. dacinus* thalli at the thorax near the base of the wing of *Bactrocera tryoni* from Queensland. (b) Queensland *Bactrocera kraussi* abdomen with thalli. (c) Same specimen, showing additional thalli on the hind leg. (d) Queensland *B. tryoni* with *S. dacinus* thalli spread on the legs and wings. (e) Lateral view of Northern Territory *Bactrocera aquilonis* with clusters of *S. dacinus* thalli on the thorax and wing base. (f) Same specimen as E, dorsal close up of thorax. (g) Solomon Islands *Bactrocera decumana* with occiput infection. (h) Solomon Islands *B. decumana* with abdomen infection. (i) Northern Territory *Bactrocera neohumeralis* with thalli clusters on the scutellum, wings, thorax, and legs

**FIGURE 3:**
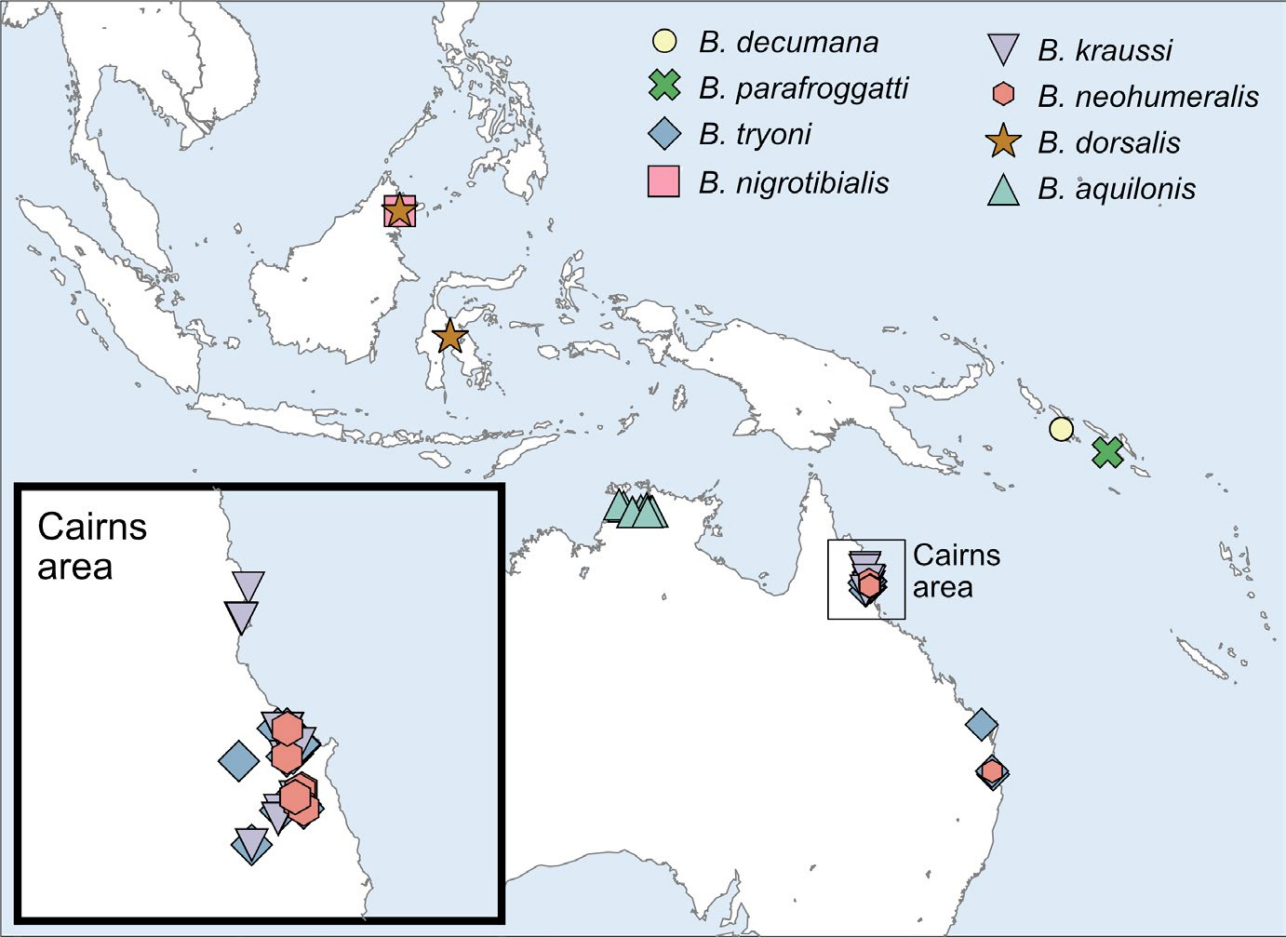
Distribution map of *Stigmatomyces dacinus* infected *Bactrocera*. *Bactrocera kraussi*, *Bactrocera tryoni,* and *Bactrocera neohumeralis* were found sympatrically infected in the Cairns area, *B. neohumeralis* and *B. tryoni* were found sympatrically infected in eastern Australia around Brisbane, and *Bactrocera dorsalis* and *Bactrocera nigrotibialis* were found sympatrically infected in Sabah, Malaysia. Otherwise all other localities contained only one infected species, but almost always along with other noninfected species (see Table S2)

Out of ~20,000 flies that we collected between 2017 and 2019, we found 197 flies infected with an ectoparasitic fungus with a uniform morphology. The fungal thalli of two infected specimens of *B. aquilonis* and two infected *B. tryoni* were slide mounted and the morphology of the fungus fits that of *S. dacinus* Thaxt. (Figure 1). *Stigmatomyces dacinus* was originally described from “*Dacus* sp. No. 2,128, Sarawak, Borneo” (Thaxter, 1918). The genus *Dacus* has since been split into *Dacus*, *Bactrocera* and *Zeugodacus*. Considering that we only found infected *Bactrocera* in field surveys, including in Borneo, it seems likely that the infected flies in the original description were what we now consider *Bactrocera*. From our surveys of ~20,000 flies, we found *S. dacinus* hosted on eight *Bactrocera* species: *B. decumana* (Drew, 1972), *B. parafroggatti* (Drew & Romig, 2001), *B. tryoni* (Froggatt, 1897), *B. nigrotibialis* (Perkins, 1938), *B. kraussi* (Hardy, 1951), *B. neohumeralis* (Hardy, 1951), *B. dorsalis* (Hendel, 1912), and *B. aquilonis* (May, 1965) (Figures 2, 3). Because some Laboulbeniales have been reported to only infect certain parts of their hosts (e.g., Rossi & Kirk‐Spriggs, 2011), we noted the body parts of *Bactrocera* that were infected. *Stigmatomyces dacinus* does not appear to be restricted in that sense; infections were observed on all body parts of the flies (Figure 2), including the eyes and wings, which likely negatively affects the fitness of the host.

### 3.1 Infection rates and pattern

**FIGURE 4:**
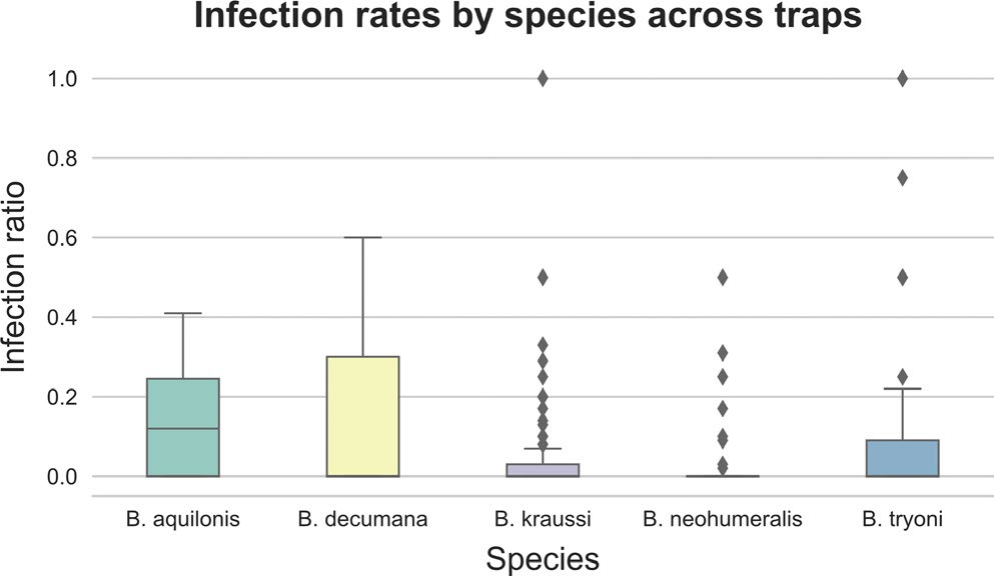
Boxplots showing the infection rates across *Bactrocera* species for which bulk samples were studied, where each bulk sample represents the flies caught in a single lure trap at a unique locality

We found only three *B. dorsalis* infected among thousands studied. Five of the infected *Bactrocera* had more than ten specimens infected; for those, we examined all trap samples in the UHIM collection—also those collected prior to 2017—to estimate infection rates (Figure 4, Table S1). Infection rates varied widely per trap sample from 0% to 100%, but infections were generally rare and the overall average infection rate was 7.86%. The median infection rate is 0% for all species except *B. aquilonis*, where it is 11%. The outlier 100% infection rates were all in traps with a single individual (Table S1). *Bactrocera kraussi*, *B. tryoni,* and *B. neohumeralis* were found sympatrically infected in the Cairns area (Australia), *B. neohumeralis* and *B. tryoni* were found sympatrically infected in eastern Australia around Brisbane, and *B. dorsalis* and *B. nigrotibialis* were found sympatrically infected in Sabah (Malaysia). It is interesting to note that infected species were found with 37 noninfected *Bactrocera* species in the same lure traps (Table S2). Some of those species were found in large numbers, such as hundreds of *B. frauenfeldi* (Schiner, 1868), *B. endiandrae* (Perkins & May, 1949), and *B. cacuminata* (Hering, 1941). This suggests that those species are systematically not infected, although it is hard to rule out that this may have been a stochastic effect as infection rates overall were low, and perhaps we did not trap the infected specimens by chance.

### 3.2 SSU sequence data

**FIGURE 5:**
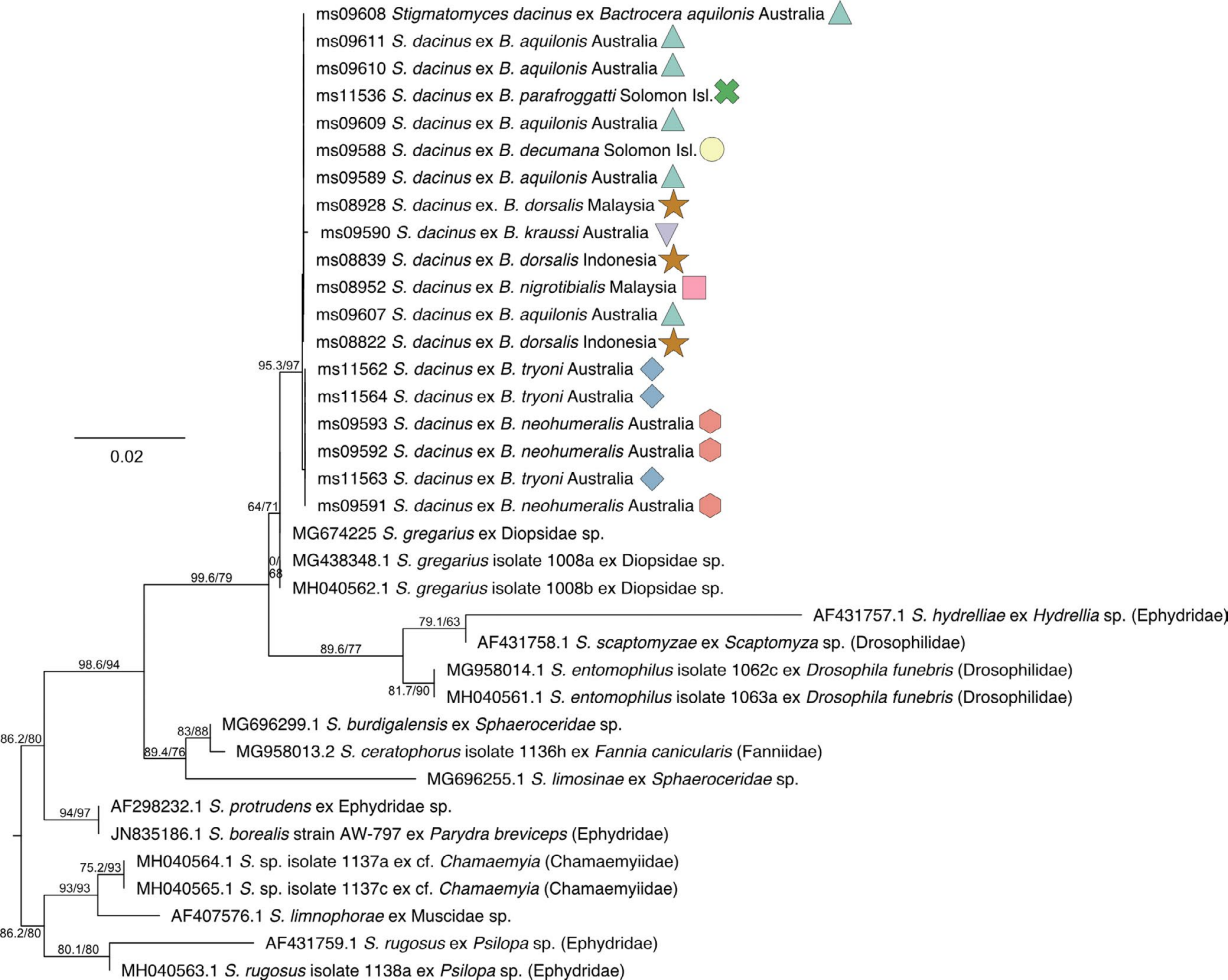
Maximum likelihood tree based on SSU sequences of *Stigmatomyces*. Taxon names starting with “ms” codes are added in this study, and other numbers refer to Genbank accession codes. For each taxon, the *Stigmatomyces* fungus species is indicated as well as the fly host taxon for the sample. The colored symbols match those in Figure 1. Support values on the branches indicate ultrafast bootstrap and SH‐aLRT values, respectively. The scale bar indicates substitutions per site

We successfully obtained small subunit (SSU) DNA sequences from 19 *S. dacinus* infections, representing all eight infected *Bactrocera* host species. The maximum likelihood tree based on SSU sequences obtained in this study combined with published *Stigmatomyces* SSU sequences (Goldmann & Weir, 2018; Haelewaters, Page, et al., 2018) shows no differentiation of *S. dacinus* between its different hosts that would suggest that they are different species (Figure 5). Although SSU is considered a conservative marker, all the species of *Stigmatomyces* included in the tree are clearly separated even when related, such as *S. entomophilus* (Peck) Thaxt. and *S. scaptomyzae* Thaxt. *Stigmatomyces protrudens* Thaxt. and *S. borealis* Thaxt. are not separated, but they are considered to be “growth forms” of the same species. The 1,363 segments of SSU that we obtained for *S. dacinus* contained two variable positions: one single nucleotide polymorphism (SNP) in the specimens that infected *B. kraussi* and a SNP in specimens that infected *B. neohumeralis* and *B. tryoni*. The variation between the overlapping larger SSU fragments is able to discern between species of *Stigmatomyces* with much larger differences, suggesting that there is a single species of fungus infecting the different *Bactrocera* species and there is no co‐evolution involved. Overall, these results support the conclusions based on morphology that all *Bactrocera* are infected by a single species of *Stigmatomyces*, but neither can rule out cryptic speciation. The closest sister species in the phylogeny is *S. gregarius* W. Rossi, isolated from a stalk‐eyed fly (Diopsidae), a host which is distantly related to *Bactrocera* (Tephritidae), but the current phylogenetic coverage includes only a fraction of the *Stigmatomyces* diversity (Hyde et al., 2019) and may lack more closely related congeners.

## 4 DISCUSSION

### 4.1 Ample hosts yet low infection rates

Although most Laboulbeniales are thought to be highly specialized on a single host species (Pedrini et al., 2015), we found no evidence for divergence in morphology or SSU DNA of *Stigmatomyces* infecting eight different species of *Bactrocera* fruit flies. This suggests that a single fungus species will infect different host species across different continents. However, not all *Bactrocera* appear susceptible; 37 congeneric species collected in the same traps as the infected hosts were never infected despite sometimes being collected in large numbers. Moreover, we found that of the infected species, five were more commonly infected (~7.8%), whereas the other three had much lower infection rates (<1%), despite the fact that some hosts in the latter category are numerous and ubiquitous (e.g., *B. dorsalis*). This suggests that there are additional factors, such as infection resistance mechanisms, determining the host range, and infection rate, which has been shown in other systems (e.g., Joop & Vilcinskas, 2016).

Similarly, low infection rates have been observed in other Laboulbeniales associated with Diptera: Bergonzo, Rossi, and Weir (2004) found 180 infected flies among 30,000 specimens collected in South America. In Laboulbeniales infecting the fruit fly genus *Anastrepha* in Costa Rica, there was a correlation between fruiting seasons and infection rates; infection rates were higher with the continuous availability of fruit and higher densities of hosts (Hedström, 1994). A study on Stalk‐eyed flies infected with Laboulbeniales found infection rates up to 100% with long‐lived (univoltine) species, and much lower infection rates with short‐lived (multivoltine) species (Rossi & Feijen, 2018). A final factor that has been proposed as influencing the infection rate is aggregation behavior: Beetles and flies that group together for mating or overwintering are more likely to have higher infection rates (De Kesel, 1993; Feijen, Martin, & Feijen, 2017; Riddick & Schaefer, 2005). *Bactrocera* are all thought to be principally multivoltine (Drew & Romig, 2013), with the number of generations depending on the seasonal availability of fruit hosts. The males of all infected species are attracted to chemicals (synomones) emitted by some flowering plants to draw flies in for pollination (Tan et al., 2002). The flies are known to congregate at such flowers, sometimes in large numbers. Detailed knowledge on the diet breadth of *Bactrocera* and their pollination behavior could shed more light on the pattern of *Stigmatomyces* infections. Based on our results and other studies, it is likely a combination of host resistance in conjunction with host ecology that ultimately determines infection rates.

### 4.2 Potential for population control?

Three of the *Bactrocera* species infected with *S. dacinus* are major agricultural pests: the Oriental fruit fly *B. dorsalis* and the sister species *B. aquilonis and* Queensland fruit fly *B. tryoni* (Blacket, Semeraro, & Malipatil, 2012; Cameron, Sved, & Gilchrist, 2010; Vargas et al., 2015). *Bactrocera dorsalis* has the largest distribution of all infected species. It is widespread throughout Southeast Asia (Vargas et al., 2015) and has invaded all of Africa and the Hawaiian islands, but is absent from Australia (Vargas et al., 2015). It is difficult to interpret the meaning of the very low infection rates in *B. dorsalis*; it could indicate stronger resistance, or higher mortality rates, where the infected flies die before they reach the traps. Although some entomopathogenic fungi have become important population regulators in agriculture (Pedrini et al., 2015), no studies published at present elucidate population control from Laboulbeniales. In addition, the fungus attacks the flies by attaching to the cuticle, but it is unclear how it obtains its nutrients (Madelin, 1966) or how it affects fitness. The current consensus is that most Laboulbeniales have a negligible effect on the fitness of their host (Haelewaters, De Kesel, et al., 2018), and a reviewer pointed out that some do not consider them to be parasitic at all. However, there are Laboulbeniales that form rhizoids that penetrate beyond the cuticle, and such species are known to negatively affect the host (Haelewaters et al., 2017). Moreover, we find it likely that all Laboulbeniales obtain nutrients from their host and that there is no conceivable benefit to the host, making them parasitic by definition regardless of any consequences to the evolutionary fitness of the host. Studies on other *Stigmatomyces* have established protocols to grow the fungi in laboratory conditions using fly wings on a nutrition substrate (Whisler, 1968). Such experiments could be a first step to testing the fitness effects of the fungus on the host. Further understanding of Laboulbeniales infection patterns and mechanisms of tephritid fruit flies could reveal their potential as biological control agents for numerous pests, and understanding the paradox of their wide host range and contrasting rarity. Understanding the mechanisms that determine infection rates will involve both studies of host resistance as well as host ecology, as the fungal parasites depend on physical contact of the hosts to be transmitted. Ultimately, this can inform on a broader scale on how host‐parasite density systems function in practice.

## CONFLICT OF INTEREST

The authors declare that they have no competing interests.

## AUTHOR CONTRIBUTIONS

**Camiel Doorenweerd:** Conceptualization (equal); data curation (equal); formal analysis (equal); funding acquisition (equal); investigation (equal); methodology (equal); project administration (equal); resources (equal); software (equal); supervision (equal); validation (equal); visualization (equal); writing – original draft (equal); writing – review and editing (equal). **Sebastian Sievert:** Data curation (equal); formal analysis (equal); writing – review and editing (equal). **Walter Rossi:** Formal analysis (equal); investigation (equal); methodology (equal); validation (equal); writing – review and editing (equal). **Daniel Rubinoff:** Conceptualization (equal); methodology (equal); resources (equal); supervision (equal); writing – review and editing (equal).

## Data Availability

DNA sequences: Genbank accessions MN935796–MN935814. DNA sequence alignment used for phylogenetic inference is available in Dryad https://doi.org/10.5061/dryad.sn02v6x1m. Sampling locations and specimen data are listed in supplemental material S1–S3, available in Dryad https://doi.org/10.5061/dryad.sn02v6x1m.
